# Good-Quality and High-Efficiency Dicing for Thick LiNbO_3_ Wafers Using Picosecond Laser Pulses

**DOI:** 10.3390/mi11010051

**Published:** 2019-12-31

**Authors:** Mingwei Lei, Wenyan Gao, Guang Li, Xinping Wu, Benhai Li, Xuefeng Wang, Junlong Wang

**Affiliations:** Beijing Institute of Aerospace Control Devices, Beijing 100094, China

**Keywords:** laser dicing, picosecond laser, dicing quality optimization, thick lithium niobate (LiNbO_3_) wafers

## Abstract

Lithium niobate (LiNbO_3_) has become popular with applications in electronics and communication industries due to its excellent electro-optical and nonlinear properties. This paper presents the influence of laser power, repetition frequency, number of subpulses, depth of each pass, and scanning velocity in picosecond laser dicing on multiple characteristics of LiNbO_3_ using the Taguchi method. By means of analysis of variance and analysis of relations between the characteristics, the optimal ps-laser-dicing parameter is obtained with good quality and high efficiency, which is applied to LiNbO_3_ products. The result indicates that picosecond laser dicing provides an alternative to machine thick LiNbO_3_ wafers with narrow kerf width, micro chipping, smooth surface, and high productivity.

## 1. Introduction

Lithium niobate (LiNbO_3_) is a crystalline dielectric material of considerable interest for its popular applications in optical communications and sensor systems [1,2,3,4]. Because of its attractive properties, such as its unique electro-optical effects, large nonlinear optical coefficients, high refractive index of 2.2, large transparent ranging from ultraviolet to infrared and excellent thermal stability, LiNbO_3_ is referred as “silicon in nonlinear optics” [5]. Traditionally, an LiNbO_3_ wafer is processed by methods of slicing, grinding, etc. However, due to high hardness, it faces serious challenges including large chipping and poor-quality surface, which would lead to the reduction of product life [6].

Ultrafast-laser material machining is promising in industrial products over conventional processing methods, without causing thermal damage. In order to improve processing precision and surface roughness, ultrafast laser machining in LiNbO_3_ has been studied intensively in recent years [7,8,9,10,11,12]. Chen et al. measured the surface-ablation threshold of LiNbO_3_, which decreases significantly along with an increase of the pulse number, until reaching a constant level due to an incubation effect [10]. Fissi et al. reported that the micromachining rate strongly depends on the operating parameters, such as laser power, scanning speed, and scanning number. Besides, laser pulse overlap around 93% for LiNbO_3_ helps to achieve optimal surface roughness [11]. Javaux’s group observed the influence of number of subpusles in burst mode and repetition frequency on transparent materials. In loose focusing configuration, the use of burst mode allows for increasing the kerf width without extra energy absorption, and the kerf width increases when the laser repetition frequency decreases [12].

According to our previous investigation on LiNbO_3_ cutting, small chipping and smooth roughness have been achieved. However, it takes hundreds of laser passes to cut such a hard material through [13]. It has been a key problem to process thick LiNbO_3_ wafers at high speed while maintaining good quality. However, laser dicing is a potential for its high throughput and good-quality edges, which is capable of suppressing the mentioned problem. Laser dicing has attracted lots of attention on processing different semiconductor materials, such as silicon [14] and glass [15,16,17,18,19]. Nevertheless, there is minimal literature investigating the effect of picosecond-laser parameters on multiple characteristics in LiNbO_3_ dicing using Taguchi method, which has been widely used in engineering to optimize the processing parameters and improve the yield and the reliability in machining a product by the means of orthogonal-array experiment design, analysis of variance (ANOVA) and signal to noise (S/N) ratio, etc.

In this paper, considering the robustness and cost-performance ratio of ultrafast laser systems, we employed a picosecond-laser experimental setup and made an orthogonal-array experimental design to study the influence of laser power, repetition frequency, number of subpulses, depth of each pass, and scanning velocity on LiNbO_3_ dicing characteristics. Using analysis of variance, the most influential processing parameter in LiNbO_3_ laser dicing was obtained. For instance, the number of subpulses plays a dominant role on chipping size and side roughness. By analysis of relations between different characteristics, kerf width, Sq roughness, and processing rate were chosen to evaluate the overall characteristics in LiNbO_3_ dicing. Finally, the optimal parameters for picosecond laser dicing were determined and applied to a LiNbO_3_ product, which was well processed with narrower width, less chipping, and smoother surface as well as efficiency increased by 10 times compared with conventional methods. Our picosecond-laser-dicing result reveals a great potential for end product of high-demanding processing.

## 2. Experimental Setup

**Figure 1:**
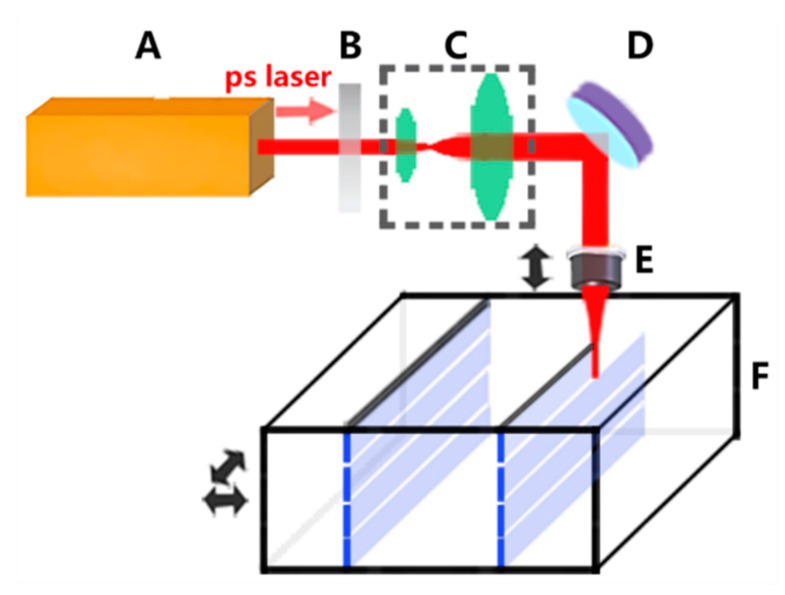
Schematic diagram of experimental setup for laser dicing. A: picosecond laser system; B: attenuator; C: beam expander; D: reflective mirror; E: objective lens; F: lithium niobate (LiNbO_3_) sample.

The experimental setup for laser dicing is illustrated in Figure 1. A commercial picosecond laser system (Rapid NX, Coherence, Santa Clara, CA, USA) delivers laser pulses with a pulse duration of 12 ps, wavelength of 1064 nm, and beam diameter of 1 mm. The laser power with the maximum of 3W is tuned by the attenuator, and the repetition frequency is various from 50 kHz to 1 MHz. The burst-mode, with up to 6 subpulses per burst, could be generated at a frequency of 40 MHz, with burst-to-burst temporal separation of 25 ns. The laser beam is expanded by a 4X-magnified expander and then focused onto an LiNbO_3_ sample with a spot diameter of 7 μm by an objective lens with a numerical aperture (NA) = 0.4. The sample is translated by the X/Y-axis motorized stages with a high scanning velocity up to 400 mm/s and the focusing position of the laser beam is controlled by the Z-axis stage with an accuracy of 5 μm. The two-side polished sample with 1-mm thickness is cleaned with alcohol before laser dicing. The process of laser dicing is to realize an intra-volume modification along the whole thickness of the sample. Because the depth of laser focus is much smaller than the sample thickness, several passes of laser dicing are required at different depths. Then a mechanical action is necessary to separate the samples along the laser-dicing path.

## 3. Results

There are many processing parameters that may affect LiNbO_3_ dicing characteristics, including the laser power, repetition frequency, number of subpulses, depth of each pass, and scanning velocity, symbolized by *p*, *f*, *n*, *d*, and *v*, respectively. In this study, these five parameters and their four levels are considered and the level numbers of 1, 2, 3, and 4 are used in the following discussion, which stand for their levels from small-to-large value. Since the number of complete experimental trials is up to 1024, an orthogonal-array experiment design is made to effectively reduce the number of experimental trials [20]. Based on the selected laser-dicing parameters and their levels, Table 1 lists the adopted experiment design, which results in only 16 sets of experiment instead of 1024.

The processing accuracy, surface quality and productivity in LiNbO_3_ wafers are of great importance due to their impact on the overall performance of the product. In this study, kerf width and chipping size in the top surface serve as processing accuracy, while the arithmetical mean roughness, the root mean square roughness, and the maximum roughness of side surface are used to evaluate surface quality. In addition, processing rate represents the productivity of laser dicing. Thus, the LiNbO_3_ dicing characteristics are estimated with kerf width, chipping size, arithmetical mean roughness, root mean square roughness, maximum roughness, and processing rate, respectively, abbreviated as *W*, *L*, *Sa*, *Sq*, *Sz*, and *R*. Here, *W* and *L* of the top surface were measured using a 500X-magnified digital microscope (DM6B, Leica, Wetzlar, Germany) with an accuracy of 0.1 μm. *Sa*, *Sq*, and *Sz* of the side surface were detected with a white surface interferometer (Zygo NewView8300, AMETEK, Ltd., New York, NY, USA). *R* was calculated with the equation as Equation (1):

(1) R=vd/t

where *v*, *d,* and *t* are scanning velocity, depth of each pass, and the sample thickness of 1 mm, respectively.

**Figure 2:**
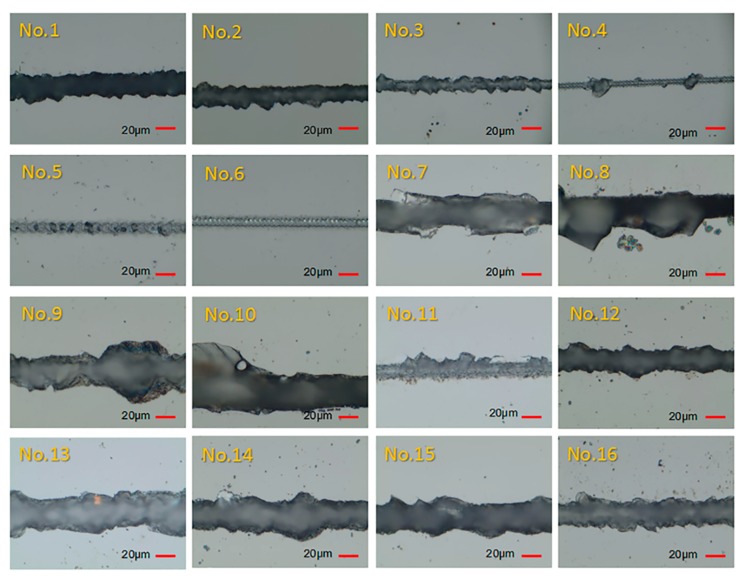
Top-surface images of laser-dicing samples under different parameters recorded with a digital microscope. The number located at each sample represents the experimental number in Table 1.

**Figure 3:**
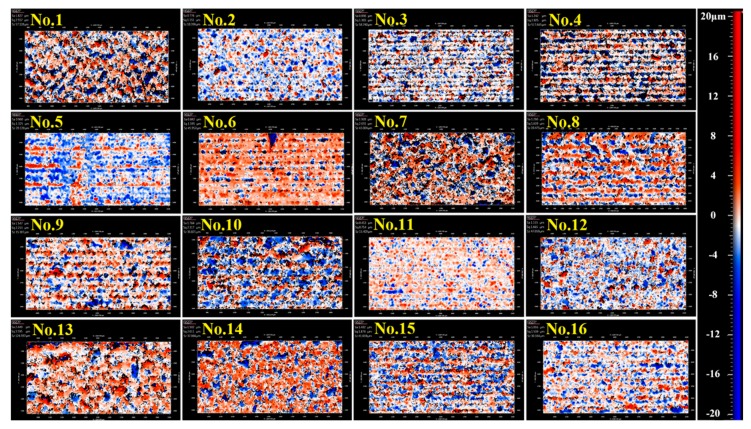
Side-surface images of laser-dicing samples under different parameters measured with white surface interferometer, wherein the red and blue points correspond to the peak and valley, respectively. The number located at each sample represents the experimental number in Table 1.

On the basis of the orthogonal-array experimental design listed in Table 1, laser-dicing experiments on the LiNbO_3_ sample were conducted. Figure 2 and Figure 3, respectively, present the images in top and side surface of the laser-dicing samples under various parameters. The numbers in the figures correspond to experimental numbers in Table 1 and indicate the experimental parameters for that particular sample. For each set of the experiment, the measurement of processing accuracy, surface roughness, and processing rate was conducted at four different points and the results were summarized in Table 2. According to Table 2, significant changes of cutting quality took place in our experimental design with a wide-ranged value of kerf width (4.6–37.9 μm), chipping size (4.8–42.2 μm), *Sa* roughness (0.45–2.45 μm), *Sq* roughness (0.71–3.60 μm), *Sz* roughness (15.41–517.65 μm), and processing rate (2–20 mm/s). From Figure 2, it is observed that bigger chippings occurring in No.8–No.10 and larger kerf width in No.13–No.15. Besides, some defects emerged in the side surface of No.1, No.4, No.7, No.10, No.13, and No.14 in Figure 3.

## 4. Discussion

**Figure 4:**
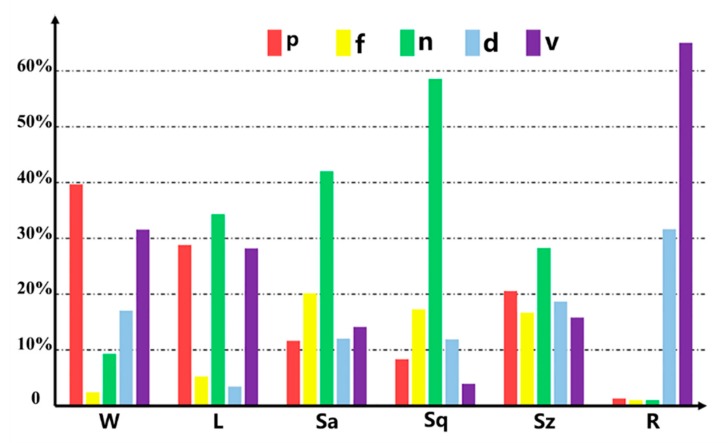
Percentages of contribution from each parameter on cutting characteristics of kerf width (*W*), chipping size (*L*), arithmetical mean roughness (*Sa*), root mean square roughness (*Sq*), maximum roughness (*Sz*), and processing rate (*R*), wherein the red, yellow, green, blue and purple bars correspond to laser-dicing parameters of laser power (*p*), repetition frequency (*f*), number of subpulses (*n*), depth of each pass (*d*), and scanning velocity (*v*), respectively.

To obtain the most significant parameter for each cutting characteristic in LiNbO_3_ dicing, analysis of variance was applied to obtain the individual significance of *p*, *f*, *n*, *d*, and *v*, which is calculated by the decomposition of the variance using a Minitab 17 statistical software (Minitab Inc., State College, PA, USA) [21]. Displayed in Figure 4, the ANOVA result reveals that the percentages of contribution from each parameter is various and dependent on the LiNbO_3_ characteristics in laser dicing. Both laser power and scanning velocity have significant effects on kerf width, and the factors account for 40% and 32% of the total variation, respectively. As for the edge chipping, number of subpulses becomes the main influencing parameter with a significant influence of 35% on chipping size. Besides, number of subpulses is still the dominant factor on *Sa*, *Sq*, and *Sz* roughness in the side surface. In terms of processing rate, scanning velocity plays the most important role with a high contribution up to 62% and is followed by the depth of each pass, which is corresponding to Equation (1). When considering promoting the characteristics of *W*, *L*, *Sa*, *Sq*, and *Sz*, the appropriate values of laser power, scanning velocity, as well as number of subpulses, must be chosen carefully because they emerge as the most influential experimental parameters in LiNbO_3_ laser dicing. The kerf width and chipping size of the top surface are directly relative to the laser-damaged area. According to previous essays [10], the diameter of the damaged area *D* depends strongly on the maximum laser fluence *F*_0_ and the material ablation-threshold *F_th_*, which is calculated by the following Equation (2):

(2) $$D^{2}\;=\;2\omega_{0}^{2}\ln\left(\frac{F_{0}}{F_{th}}\right)$$

where *ω*_0_ is the laser beam radius. Increasing the laser power tends to increase the maximum laser fluence *F*_0_, while increasing the scanning time tends to decrease the material ablation-threshold *F_th_* because of an increase in the pulse number applied to the surface. Thus, laser power and scanning velocity have significant effects on both kerf width and chipping size. Besides, compared with a single laser pulse, the absorption coefficient under burstmode laser pulse would increase due to the accumulation of laser-induced modification. For simplicity, when using burstmode laser pulse with *n* subpulses, the modified absorptivity $\alpha_{n}$ can be estimated by Equation (3):

(3) $$\alpha_{n}=\alpha_{1}+\left(n-1\right)\Delta \alpha$$

where $\alpha_{1}$ is the initial absorptivity and Δα is the increase of absorptivity induced by subsequent subpulse in burstmode laser. As a result, more defects in the intra volume of the sample are easy to appear in burst-mode laser machining and the side surface becomes rougher with a larger number of subpulses. It is recommended that better dicing quality can be obtained when using a lower laser power and a larger scanning velocity and smaller number of subpulses, but the laser power should be larger than the ablation threshold of LiNbO_3_ material in order to cause laser-induced modification. On the other side, depth of each pass is less important for LiNbO_3_ dicing quality, which implies processing rate can be enlarged reasonably by means of increasing the depth of each pass while maintaining a good quality of LiNbO_3_ dicing.

**Figure 5:**
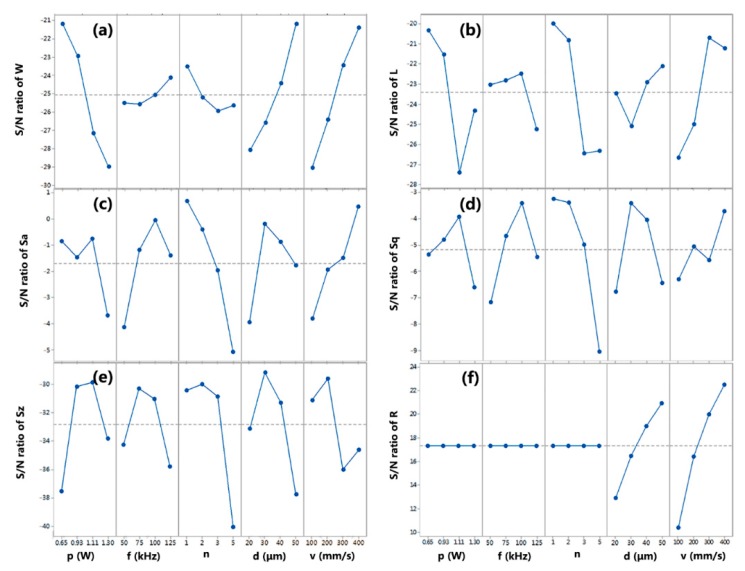
Signal-to-noise effects for laser dicing parameters of *p*, *f*, *n*, *d* and *v* on LiNbO_3_ cutting characteristics of (**a**) *W*, (**b**) *L*, (**c**) *Sa*, (**d**) *Sq*, (**e**) *Sz* and (**f**) *R*.

To investigate how the process parameters affect the cutting characteristics in laser dicing, the signal-to-noise (S/N) ratio is used to measure the variations of experimental result. The smaller values of *W*, *L*, *Sa*, *Sq*, *Sz* are desired, However, the larger value of *R* indicates the better performance. S/N ratios of *W*, *L*, *Sa*, *Sq*, and *Sz* depending on the criterion of smaller-the-better are calculated by Equation (4):

(4) $$S/N\;=\;-10\log\left(\frac{1}{n}\sum_{i=1}^{n}y_{i}^{2}\right)$$

while the S/N ratio of *R* depending on the criterion of larger-the-better is given by Equation (5):

(5) $$S/N\;=\;-10\log\left(\frac{1}{n}\sum_{i=1}^{n}\frac{1}{y_{i}^{2}}\right)$$

where *y_i_* is the observed response value at the *i*-th trail and n is the number of observations [22]. Based on the larger S/N ratio criterion, the optimal level of the process parameters is the level with the highest S/N ratio in Figure 5. For instance, the processing parameters of laser power at level 1, repetition frequency at level 4, number of subpulses at level 1, depth of each pass at level 4, and scanning velocity at level 4 yield the best result of kerf width. As a result, the value of *W* is the smallest when using the parameter combination of *p*1-*f*4-*n*1-*d*4-*v*4, which is corresponding to the mentioned theoretical discussion. Similarly, it can be concluded that the optimal parameter combination of levels for the smallest *L*, and the lowest *Sa*, *Sq*, *Sz*, are *p*1-*f*3-*n*1-*d*4-*v*3, *p*1-*f*3-*n*1-*d*2-*v*4, *p*3-*f*3-*n*1-*d*2-*v*4, *p*3-*f*2-*n*2-*d*2-*v*2, respectively. However, the optimal combination for the fastest *R* is *d*4-*v*4, because processing rate only depends on scanning velocity and depth of each pass based on Equation (1). Since the best parameter combination for the six characteristics are different from each other, a tradeoff between processing accuracy, surface roughness, and processing rate in laser dicing is necessary.

**Figure 6:**
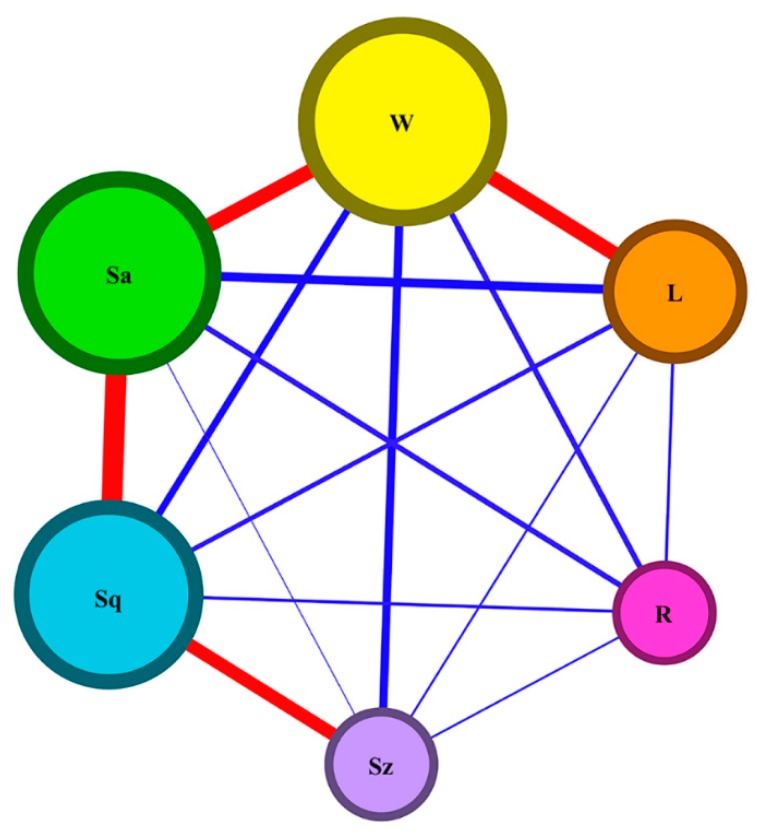
Analysis of relations between the cutting characteristics of *W*, *L*, *Sa*, *Sq*, *Sz*, and *R*. Red and blue lines indicate that their relation strength is large and small, respectively. The thickness of the line indicates the value of the correlation coefficient of the two characteristics and the size of circle indicates the sum of coefficients related.

In order to determine the optimal parameter combination for the overall characteristic, the above six characteristics have to be degenerated and simplified. The analysis of mutual relations between kerf width, chipping size, *Sa*, *Sq*, *Sz* of side roughness, and processing rate is applied with Pearson’s correlation coefficient. The coefficient is calculated by dividing the covariance of the two variables by the product of their standard deviations. When the coefficient is bigger than 0.5, it means the relation strength is large. The analysis result is shown in Figure 6, wherein the thickness of the line indicates the value of the correlation coefficient and the size of circle indicates the sum of coefficients related. Because the correlations between processing rate and other characteristics are too weak, R must be an additional consideration. Fortunately, the correlation coefficients between *W*, *L*, and *Sa*, as well as those between *Sq*, *Sa*, and *Sz*, are bigger than 0.5. Thus, *W* is strongly relative to *L* and *Sa*, meanwhile *Sq* is significantly associated with *Sa* and *Sz*. It can be concluded that a good performance of *L*, *Sa*, and *Sz* would be achieved with the optimal values of *W* and *Sq*. As a result, kerf width, *Sq* roughness, and processing rate are selected as the representative characteristics in LiNbO_3_ laser dicing. With the help of Figure 4, laser power is the most important for *W*, repetition frequency and number of subpulses are the most important for *Sa*, and depth of each pass and scanning velocity are the most important for *R*. According to Figure 5, laser power at level 1 yields the optimal result of *W*, repetition frequency at level 3 and number of subpulses at level 1 yield the optimal result of *Sq*, and depth of each pass at level 4 and scanning velocity at level 4 yield the best result of *R*. Based on the above analysis, the optimized parameter combination of levels for overall characteristic are determined as *p*1-*f*3-*n*1-*d*4-*v*4. Therefore, a low laser power of 0.65 W, a high repetition frequency of 100 kHz, a small number of subpulses of 1, a large depth of each pass of 50 μm, and a fast scanning velocity of 400 mm/s are recommended to obtain better laser-dicing lithium niobate with high processing accuracy, smooth surface roughness, and large productivity.

**Figure 7:**
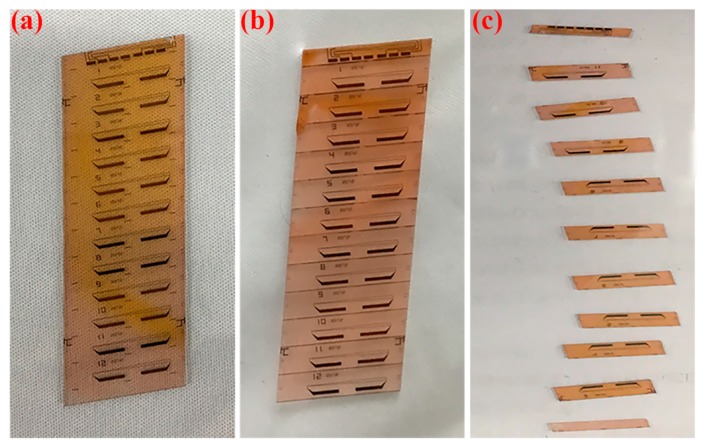
Images of LiNbO_3_ wafer (**a**) before and (**b**) after laser dicing and (**c**) the separated electro-optic waveguide modulators.

**Figure 8:**
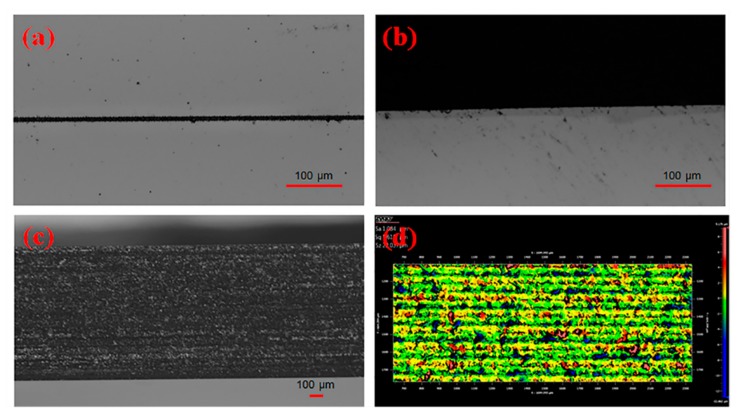
Images of the top surface of the LiNbO_3_ modulator (**a**) before and (**b**) after the breaking process. Images of side surface detected by digital microscopy (**c**) and white surface interferometer (**d**) were also attached.

Shown in Figure 7a, electro-optic waveguide modulators on a 1-mm thick LiNbO_3_ wafer were applied in picosecond laser dicing with the optimal parameters of *p*1-*f*3-*n*1-*d*4-*v*4, which are the products used in fiber optic gyroscope. To avoid the electro-optic waveguide modulators being polluted, photoresist was used to protect them from debris during machining, and laser dicing started from the reverse surface of the wafer. The LiNbO_3_ wafer was fixed on the X/Y-axis stages and processed in the experimental setup illustrated in Figure 1. The picosecond laser system was operated in single pulse mode with laser power as 0.65 W and repetition rate as 100 kHz. In addition, the wafer was fed through the laser beam with a speed of 400 mm/s, and 20 passes were taken with 50-μm depth of each pass to cover the wafer thickness. Figure 7b presents the image of the wafer after lasing dicing. Then, under mechanical stress, the wafer was divided into a dozen electro-optic waveguide modulators in Figure 7c. Figure 8a,b show the top surface of the wafer before and after the breaking process, meanwhile kerf width and chipping size are small as 7.5 μm and 5.8 μm, respectively. Less defects at the side surface could be identified in Figure 8c, and it was further examined by white surface interferometer with an *Sa* of 1.08 μm, *Sq* of 1.61 μm, and *Sz* of 22.04 μm in Figure 8d. Moreover, it consumed about 3 min per wafer dicing instead of half an hour when using conventional methods, which implied that the processing rate of laser dicing was increased by an order of magnitude. Besides, the best-performance sample in Table 2 is No.5 using the processing parameter combination of *p*2-*f*1-*n*2-*d*3-*v*4 with 9.1 μm (*W*), 6.3 μm (*L*), 0.96 μm (*Sa*), 1.33 μm (*Sq*), 28.12 μm (*Sz*), and 16 mm/s (*R*). Because we decreased the laser power, reduced the number of subpulses and increased the depth of each pass, the final application was better than No.5 sample in Table 2 with a narrower kerf width, a smaller chipping, and a higher productivity. Thus, the method of laser dicing is promising in processing the thick wafer, which needs both good quality and high efficiency.

## 5. Conclusions

In conclusion, LiNbO_3_ dicing experiments with picosecond laser pulses were investigated by an orthogonal-array experimental design. Based on analysis of variance, number of subpulses, scanning velocity, and laser power are the most influential experimental parameters on LiNbO_3_ cutting characteristics. Using analysis of relations, we obtained the optimal combination with *p*1-*f*3-*n*1-*d*4-*v*4 for the overall dicing characteristic. LiNbO_3_ products were demonstrated and achieved a narrow kerf width of 7.5 μm, a small chipping of 5.8 μm, a smooth surface of 1.08 μm (*Sa*), and a high productivity of 20 mm/s after our laser processing. The high-quality result reveals that our developed picosecond-laser dicing has potential in machining LiNbO_3_ wafers for electronics and communication applications, in which processing precision, surface roughness, and productivity are strictly demanded.

## Figures and Tables

**Table 1:** Orthogonal-array experimental design of laser-dicing parameters.

Exp. Number	Laser Power (*p*/W)	Repetition Frequency (*f*/kHz)	Number of Subpulses (*n*)	Depth of Each Pass (*d*/μm)	Scanning Velocity (*v*/mm/s)
1	0.65	50	1	20	100
2	0.65	75	2	30	200
3	0.65	100	3	40	300
4	0.65	125	5	50	400
5	0.93	50	2	40	400
6	0.93	75	1	50	300
7	0.93	100	5	20	200
8	0.93	125	3	30	100
9	1.11	50	3	50	200
10	1.11	75	5	40	100
11	1.11	100	1	30	400
12	1.11	125	2	20	300
13	1.30	50	5	30	300
14	1.30	75	3	20	400
15	1.30	100	2	50	100
16	1.30	125	1	40	200

**Table 2:** Experimental results for lithium niobate (LiNbO_3_) dicing characteristics under different parameters.

Exp. Number	Kerf Width (*W*/μm)	Chipping Size (*L*/μm)	Arithmetical Mean Roughness (*Sa*/μm)	Root Mean Square Roughness (*Sq*/μm)	Maximum Roughness (*Sz*/μm)	Processing Rate (*R*/mm/s)
1	22.5	9.9	1.83	2.56	57.13	2
2	17.2	10.6	0.78	1.15	18.40	6
3	9.8	9.2	0.84	1.37	58.74	12
4	4.6	12.1	1.24	2.93	517.65	20
5	9.1	6.3	0.96	1.33	28.12	16
6	6.6	4.8	0.84	1.60	45.96	15
7	24.7	18.2	1.93	2.63	43.00	4
8	26.2	36.8	1.27	1.64	19.48	3
9	19.8	33.0	1.55	2.21	35.39	10
10	37.9	42.2	1.76	2.32	36.82	4
11	14.8	13.5	0.45	0.71	15.41	12
12	24.3	15.9	1.16	1.67	47.06	6
13	31.0	19.7	2.45	3.60	124.60	9
14	30.5	17.2	1.50	2.01	37.07	8
15	28.8	13.8	1.40	1.87	41.66	5
16	22.9	15.7	1.06	1.54	30.33	8
